# Editorial: Protein modifications in epigenetic dysfunctional diseases: mechanisms and potential therapeutic strategies

**DOI:** 10.3389/fcell.2023.1216637

**Published:** 2023-05-15

**Authors:** Jing Ji, Aixin Jing, Ting Geng, Xinhui Ma, Wei Liu, Bin Liu

**Affiliations:** ^1^ Key Laboratory of Marine Pharmaceutical Compound Screening, College of Pharmacy, Jiangsu Ocean University, Lianyungang, China; ^2^ School of Molecular Sciences and Biodesign Center for Applied Structural Discovery, Arizona State University, Tempe, AZ, United States

**Keywords:** DNA methylation, post-translational modifications (PMTs), epigenetic regulation, epigenetic mechanism, epigenetic-related disorders

## Introduction

Epigenetic regulation refers to the chemical modifications that affect the chromatin state and regulate gene expression without altering the DNA sequence. It consists of various molecular mechanisms, including DNA methylation, histone modifications, chromosomal remodeling, and non-coding RNA regulation. Abnormal epigenetic regulation is closely associated with the occurrence and development of many diseases (Tsankova et al., 2007; Orioli and Dellambra, 2018; Lu et al., 2020), with DNA methylation and post-translational modifications (PTMs) being important mechanisms. DNA methylation is a chemical modification in which a methyl group (CH3) is covalently attached to cytosine nucleotides in DNA. This modification can silence genes and affect gene expression. Abnormal DNA methylation in many diseases leads to changes in gene expression levels, affecting cell growth, differentiation, and apoptosis, ultimately promoting disease development (Greenberg and Bourc’his, 2019). Post-translational modifications refer to the process by which the structure and function of a protein molecule are changed through chemical modifications after protein synthesis. These modifications include phosphorylation, ubiquitination, and SUMOylation, among others. Abnormal post-translational modifications in many diseases lead to changes in protein structure and function, affecting cell metabolism, signaling, and apoptosis, among other processes, ultimately promoting disease development (Vucic et al., 2011). Therefore, in-depth research on the role of abnormal epigenetic regulation in diseases can help to deepen our understanding of the mechanisms underlying disease occurrence and development, providing new ideas and methods for the prevention and treatment of related diseases.

This Research Topic aims to uncover new insights into the roles of DNA methylation and PTMs involved in multiple epigenetic-related disorders, including tumors, ischemic stroke, atherosclerosis, and coronary heart disease (CHD). Such discoveries hold the potential to elucidate the underlying mechanisms driving the onset, progression, and therapeutic opportunities for these diseases.

### DNA methylation in epigenetic-related disorders

DNA methylation is a widely studied field in cancer research due to its significant role in the development and progression of the disease. DNA methylation can promote or inhibit cancer development by altering gene expression levels. Specific subtypes of cancer have demonstrated distinct DNA methylation patterns, which can serve as biomarkers for diagnosis or predicting treatment response. Zeng et al. conducted a comprehensive review of studies investigating the relationship between DNA methylation and the etiology, diagnosis, treatment, and prognosis of gastric cancer. Huang et al. created a gene signature and regulatory network related to enhancers, utilizing transcriptome and methylation data for predicting the survival of patients with lung adenocarcinoma.

CHD, characterized by myocardial ischemia and myocardial infarction, arises from inadequate blood supply to the coronary arteries. Abnormal DNA methylation has been shown to affect the expression of genes involved in vascular function regulation, inflammatory factors, and blood clotting (Xia et al., 2021). These aberrant DNA methylation changes may exacerbate the development and progression of CHD. Zhao et al. proposed the use of blood-based F2RL3 methylation as a potential biomarker for CHD, particularly in older individuals or those with a history of myocardial infarction. Combining F2RL3 methylation with conventional risk factors could be an effective approach for early-stage evaluation of CHD.

Ischemic stroke occurs when the brain receives insufficient blood supply, resulting in tissue damage. Research indicates a close relationship between abnormal DNA methylation patterns and the occurrence and prognosis of stroke. Altered DNA methylation patterns can influence the expression of stroke-related genes, such as neuroprotective factors and inflammatory factors, thereby affecting brain tissue damage and repair processes (Choi et al., 2022). Sun et al. presented findings of genome-wide alterations in DNA methylation in patients with ischemic stroke compared to controls. They identified 462 functional differentially methylated positions (DMPs) corresponding to 373 annotated genes. Notably, hypomethylated sites were eight times more abundant than hypermethylated sites in ischemic stroke cases, highlighting the predominant role of hypomethylation in this condition.

Atherosclerosis, a chronic inflammatory disease characterized by the buildup of lipid-rich plaques in arterial walls. Aberrant DNA methylation patterns have been observed in various cell types implicated in atherosclerosis, including endothelial cells, smooth muscle cells, and immune cells (Dai et al., 2022). Xu et al. conducted an analysis of GEO data obtained from 15 atherosclerotic and paired healthy tissues. They systematically screened the entire genome and identified a total of 110,695 differentially methylated sites (DMPs) and 918 differentially methylated regions (DMRs). Additionally, they discovered six genes exhibiting significant methylation differences in the CpG islands of the promoter regions, encompassing 49 DMPs. Notably, they also observed a notable increase in monocyte infiltration levels within atherosclerotic (AS) tissues. These findings provide valuable insights into potential DNA methylation-related biomarkers and shed light on the involvement of monocytes in early-stage atherosclerosis.

### PTMs in epigenetic-related disorders

As an important form of PTMs, SUMOylation modifications play a critical role in various biological processes, including the development and progression of cancer (Han et al., 2018). Chen et al. investigated the SUMOylation patterns in lung adenocarcinoma by employing unsupervised consensus clustering based on the expression of SUMOylation regulatory genes. Their findings demonstrated that these SUMOylation patterns effectively differentiate the tumor microenvironment characteristics of lung adenocarcinoma, particularly the status of immune cell infiltration. Additionally, they developed a SUMOylation score that enables the assessment of the relationship between SUMOylation and immune cell crosstalk. This score holds significant prognostic value and can be utilized to predict the response to immunotherapy and chemotherapy in patients with lung adenocarcinoma.

## Conclusion

The present Research Topic collected some interesting papers and revealed better understanding the mechanisms of multiple epigenetic-related disorders. We hope further researches about this Research Topic will be continued, and will contribute to the clinical transformation.
